# Research on Dynamic Routing Mechanisms in Wireless Sensor Networks

**DOI:** 10.1155/2014/165694

**Published:** 2014-04-10

**Authors:** A. Q. Zhao, Y. N. Weng, Y. Lu, C. Y. Liu

**Affiliations:** ^1^School of Computer and Information Technology, Beijing Jiaotong University, Beijing, China; ^2^Beijing Subway Operation Technology Centre, Beijing, China

## Abstract

WirelessHART is the most widely applied standard in wireless sensor networks nowadays. However, it does not provide any dynamic routing mechanism, which is important for the reliability and robustness of the wireless network applications. In this paper, a collection tree protocol based, dynamic routing mechanism was proposed for WirelessHART network. The dynamic routing mechanism was evaluated through several simulation experiments in three aspects: time for generating the topology, link quality, and stability of network. Besides, the data transmission efficiency of this routing mechanism was analyzed. The simulation and evaluation results show that this mechanism can act as a dynamic routing mechanism for the TDMA-based wireless sensor network.

## 1. Introduction

In recent years, with the rapid progress of the WSN (wireless sensor network) [1, 2], wireless devices have been widely deployed in industrial environments to improve the efficiency of the industrial process. The corresponding wireless networking standards and protocols have also emerged one after the other. The most widely applied standard in WSN is the WirelessHART (highway addressable remote transducer) [3, 4] which is a variant of HART with a simple, reliable, and secure communication between wireless devices for an industrial automated process. However, WirelessHART does not provide a clear strategy in relation to dynamic routing, which is important for the reliability and robustness of the industrial wireless network applications. In the graph routing protocol applied in WirelessHART, each node only has two parents. If these two parents are both blocked, the node will be totally separated from the network which has been proved to be an embarrassment.

In this paper, a dynamic routing strategy is proposed by evaluating and improving the CTP (collection tree protocol) [5, 6] to make it work as a solution to the dynamic routing mechanism for IWSN (industrial wireless sensor network). The evaluation of this mechanism is mainly concerned with three aspects: the time relating to generating topology, the link quality, and the stability of the topology. Through these three aspects, the rationality of this design is apparent.

## 2. Related Work

### 2.1. WirelessHART

A wireless sensor network consists of autonomous sensors used to collect information and to cooperatively pass their data through the network to a main location. WirelessHART is a sensor mesh communication system which operates in the 2.4 GHz ISM (industrial scientific medical) band at the physical layer and uses the TDMA (time division multiple access) at the data link layer. In the WirelessHART data link layer, time is separated into equal time slots and a certain number of slots constitute a super frame. In order to avoid conflict, each connection is arranged into a certain time slot with the help of TDMA technology.

### 2.2. Collection Tree Protocol

CTP is a tree-based protocol which is designed for relatively low traffic rates in WSN. Some nodes in a network can advertise themselves as the tree roots of this network. Other nodes generate routes to roots using a routing gradient.

CTP uses ETX (expected transmissions) as the routing gradient [7, 8]. The ETX of a root is 0. The ETX of a node is the ETX of its parent plus the ETX of the link to its parent. A node should always choose the node as its parent with the lowest ETX.

In the CTP, each node maintains a neighbor table with a size of 10. Each node broadcasts beacon packets periodically which contain their own routing information such as the ETX and parent to do the routing job. If a node finds that its parent is congested or there is a better parent, it will evict the current parent and choose the best neighbor in the neighbor table as its new parent. In the CTP, there is a parameter named* ETX_Threshold* which is used to work as a threshold when choosing the best neighbor. A neighbor with a link ETX value larger than the* ETX_Threshold* cannot be chosen as the best neighbor.

### 2.3. Trickle Algorithm

Trickle is a self-regulating algorithm for the propagation and maintenance of the code updates in a wireless sensor network [9, 10]. Propagation should be as quick as possible, while the maintenance cost should be as small as possible. The basic mechanism of the Trickle Algorithm is that, periodically, a node transmits its information if it has not heard other nodes transmit the same thing for a certain length of time in a manner similar to “polite gossip” [11, 12]. Instead of flooding the network with packets, Trickle Algorithm controls the sending rate to reduce the amount of packets in the network.

Formally, each node maintains a counter *c*, a threshold *k*, and a constant time point *t* in the range of [0, *τ*]. When a node hears metadata, which is the same as its own, it increases *c*. At time *t*, the node broadcasts a summary of its own program if *c* < *k*. When the interval of *τ* expires, *c* is reset to zero and *t* is set to a new random value in the range of [0, *τ*].

Parameter *τ* in Trickle Algorithm which is applied in the CTP is in the range of [*τL*, *τH*]. When nodes hear nothing new, they double the interval *τ*.

## 3. Dynamic Routing Mechanisms

A dynamic routing protocol is important for the network's reliability and robustness which is essential in industrial wireless sensor networks. However, the existing routing protocols such as graph routing in WirelessHART do not provide a dynamic routing mechanism. In this paper, two possible mechanisms are proposed as optional solutions for dynamic routing in IWSN.

The main method of the dynamic routing mechanism involves adding a CSMA (carrier sense multiple access) period into the TDMA by occupying one or several time slots in the TDMA. The reason why a CSMA period has been added into the TDMA is that a distributed period is required in order to complete some jobs, which prove not to be possible within the TDMA period. In the TDMA, everything has already been fixed. Every node knows its parent and which time slot is assigned to which link. However, the TDMA is unable to determine whether there are an unknown number of devices and thus the requirement for a distributed competing period to complete, for example, the synchronization and topology generation.

Generating and maintaining topology and dissemination are performed by broadcasting beacon packets in a CSMA period. While in the TDMA period, the nodes stop broadcasting beacon packets and transmit data packets. Thus, the CSMA period is required to be as short as possible and, at the same time, the link quality should be as good as possible.

**Figure 1:**
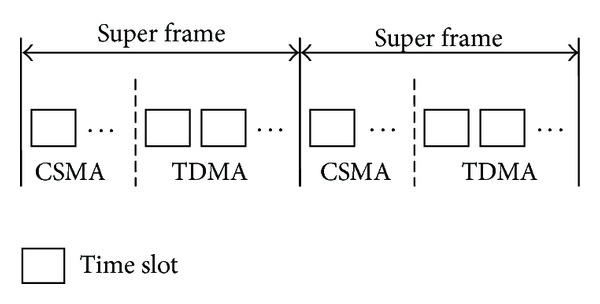
Dynamic routing mechanism without setup period.

If the CTP can generate a topology with relatively good link quality in a short period of time, the dynamic routing mechanism shown in Figure 1 can be chosen; that is, only several time slots of each super frame are needed to be assigned to the CSMA period in order to generate and maintain the topology.

**Figure 2:**
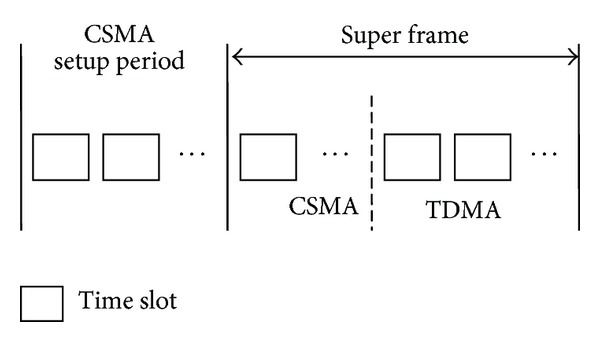
Dynamic routing mechanism with setup period.

If the CTP cannot generate a sufficiently good topology in a short period of time, then a setup period should be added which is based on the initial CSMA so as to generate the topology and disseminate the link scheduling information. In the next super frames, several time slots are assigned to the CSMA period in order to maintain the topology. This mechanism is based on an assumption that the topology generated in the setup period is relatively stable which means that the nodes will not change their parents frequently.  Figure 2 shows the dynamic routing mechanism with a setup period.

## 4. Simulations and Evaluation

In this section, the CTP-based dynamic routing mechanisms were simulated with simulator TOSSIM [13, 14] and then they were evaluated in relation to multiple aspects. According to the evaluation result, a better mechanism from two possible mechanisms was chosen and this was proposed as a solution to the dynamic routing strategy for an IWSN based on the WirelessHART standard.

TOSSIM is a discrete event simulator for TinyOS sensor networks [15, 16]. It incorporates realistic signal propagation and a noise model derived from real-world deployments. TinyOS is the first open-source operating system which is specially designed for wireless sensors and which is lightweight and makes the building of a wireless sensor network easier.

In the simulations, a uniform topology with 100 nodes in a 10 × 10 square has been adopted and a Meyer-short noise has been added into the topology [17]. After the simulation results have been output into the data log file, MATLAB can be used to process the data and to enable a visualization of the results to be made.

The evaluation of the CTP-based dynamic routing mechanisms will be based on three aspects: the time for generating the topology, link quality, and stability of the network. The parameter *τL* in the Trickle Algorithm applied in the CTP affects the time of generating the topology. The parameter* ETX_Threshold* in the CTP affects the link quality and also the time for generating the topology. If *T* and *Q* are used to represent the time of generating the topology and the link quality, respectively, then

(1) T=f(τL,ETX_Threshold),

(2) Q=g(ETX_Threshold).

Thus, the parameters *τL* and* ETX_Threshold* will be changed to seek a balance between the time of generating the topology and the link quality.

The stability of the network will determine whether the mechanism, with a setup period, can be applied. If there are too many parent changes after the topology is generated, the CSMA period should, correspondingly, be sufficiently long for the broadcasting of the beacon packets. In that case, it is not possible to apply the mechanism as a solution to the dynamic routing in the IWSN.

### 4.1. Time of Generating the Topology

Firstly, the definitions about when it is possible to identify that a node has joined into the topology and when the whole topology has been generated should be provided. It is considered that a node has joined into the topology the first time it finds its parent. When all of the nodes have found their parents, the topology is considered to have been generated.

The time of generating the topology is mainly affected by a parameter *τL* maintained in the Trickle Algorithm. The *τL* applied in the original CTP is quite large because, in the original CTP, the beacon packets and the data packets are transmitted at the same time. In order to guarantee the packet delivery ratio of the data packets, the CTP has to slow down the sending rate of the beacon packets in order to reduce the number of conflicts and to save the bandwidth. However, in the design of dynamic routing mechanisms, the CSMA period is only used to transmit the beacon packets. The demand in this case is to generate the topology as rapidly as possible regardless of the sending rate of the beacon packets. Thus, the *τL* is changed to small values to determine how fast the topology can be generated.

Two kinds of simulation experiments are presented in this section. The first is to find the *τL* with which CTP can generate topology in the shortest time; that is, in the case of a fixed* ETX_Threshold* in (1), the optimal *τL* is found out to let *T* be minimal. Secondly, the time for generating the topology is determined and consideration is given to the link quality obtained with the constant *τL*; that is, in the case of the optimal *τL* in (1), the range of* ETX_Threshold* is found out to let *T* be reasonable.

**Figure 3:**
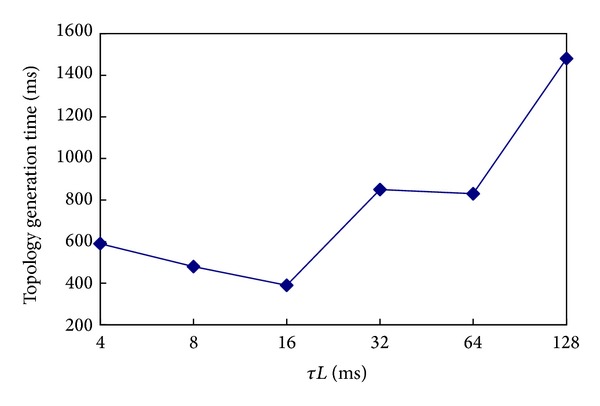
Time of generating the topology.

In the first experiment, the* ETX_Threshold* was set to a constant value and the *τL* was chosen to be 128 ms, 64 ms, 32 ms, 16 ms, 8 ms, and 4 ms. For each value, the experiment was repeated 100 times. The results are shown in Figure 3.

As can be seen in Figure 3, the *x*-axis represents the value of *τL* and the *y*-axis represents the time in which the topology is generated in 95% of the cases. It is obvious that when the value of *τL* is 16 ms, the CTP can generate the topology the fastest. The reason why it takes a long time to generate the topology with a large *τL* is because the beacon packets are transmitted once during a long time and the routing information cannot be updated quickly. When the *τL* is too small, too many beacon packets are transmitted in a certain time which will lead to a large number of conflicts. Thus this causes the nodes to be unable to hear each other. This is the reason why the time for generating the topology is longer when the *τL* is smaller.

The same experiments were repeated in the case of changing the value of parameter* ETX_Threshold*. The results show that when* ETX_Threshold* is set to a different value, the shortest time for generating the topology will change, but the condition resulting in the shortest time will not change, that is, when the parameter *τL* is set to 16 ms.

In this experiment, the time of generating the topology is 385 ms. It is quite a small value because the* ETX_Threshold* was set to be a very large value which may cause a very bad link quality. Next, we will study the time of generating the topology on the base of the link quality.

In the second experiment, the value of the* ETX_Threshold* was now changed in order to find the time for generating the topology when *τL* is a constant, namely, 16 ms. The value of the* ETX_Threshold* will be changed within the range (50, 40, 30, 20) to determine the minimum time for generating the topology with a relatively good link quality. For each value, the experiment was repeated 100 times.

The* ETX_Threshold* can affect the time of generating the topology because when a node initially chooses the best neighbour as its parent, the* ETX_Threshold* places constraints in relation to the choice of the best neighbour. A larger* ETX_Threshold* may cause a node to find its best neighbour in its neighbour table more rapidly.

**Figure 4:**
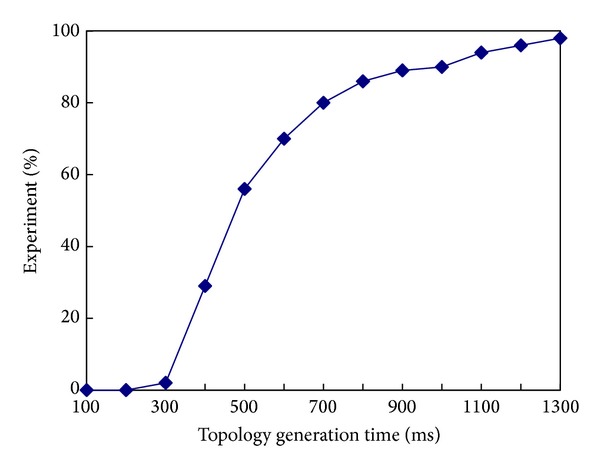
Distribution of time of generating topology when* ETX_Threshold* = 50.

Figure 4 shows the time for generating the topology 100 times when the* ETX_Threshold* is 50. The *x*-axis represents the time for generating the topology, and the *y*-axis represents the percentage of the experiment. As can be seen in Figure 4, in 95% of the cases, the CTP can generate the topology in 1176 ms.

For the next few experiments when the* ETX_Threshold* is 40, 30, and 20, the same method is applied. The experiment results are shown in Table 1.

As can be seen from the results of the experiments above, the time for generating the topology is too long when the* ETX_Threshold* is 20 and 30. It is not possible to have such a long time for the IWSN and thus some experiments must now be conducted for the values 40 and 50.

### 4.2. Link Quality

As is described in the related work, the CTP uses ETX as the routing gradient to estimate the link quality. By determining the relationship between ETX and PDR (packet delivery ratio), this will provide a clearer visualisation with regard to link quality. At the present time, no document exists which explains the relationship between ETX and PDR. In this paper, a method is designed to calculate the ETX according to the PDR. The ETX is calculated by means of the PDR using an equation named EWMA (exponential weighted moving average) [18] more times. The detailed process is as follows.

When the total number of transmitted packets represented as* data_total* reaches a fixed window size,* data_total* and the number of successfully received packets represented as* data_success* are used to update the measured value of ETX represented as* M_ETX*. The equation is

(3) $$\begin{array}{c} M\_ETX_{i}=\left(\frac{data\_total_{i}}{data\_{success}_{i}}-1\right)*10. \end{array}$$

In order to reduce the jitter of link quality, EWMA is applied to calculate the estimated value of ETX represented as* E_ETX*. The equation is

(4) $$\begin{array}{c} E\_ETX_{i}=\alpha *E\_ETX_{i-1}+\left(1-\alpha \right)*M\_ETX_{i}. \end{array}$$

In this experiment the *τL* was set to 16 ms and the* ETX_Threshold* was changed to between 40 and 50 to compare the link quality.

**Figure 5:**
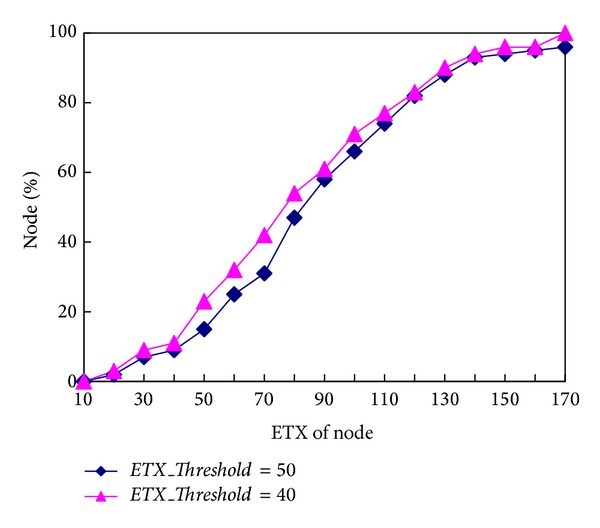
Distribution of node ETX when* ETX_Threshold* is 50 and 40.

Figure 5 shows the link quality of the 100 nodes when the* ETX_Threshold* is 50 and 40. The *x*-axis represents the node ETX, and the *y*-axis represents the percentage of nodes. In Figure 5, the blue curve represents the experiment results when* ETX_Threshold* = 50. The experiment results when* ETX_Threshold* = 40 are shown also in Figure 5 as red curve.

As can be seen in Figure 5, the link quality is a little better when the* ETX_Threshold* is 40. However, the time of generating the topology is shorter when the* ETX_Threshold* is 50 as shown in Table 1. The time for generating the topology is 353 ms longer when the* ETX_Threshold* is 40 and this value is acceptable so the value of the parameter* ETX_Threshold* should be 40.

However, a problem now arises, namely, that the time for generating the topology at 1529 ms is too long for a super frame. Thus, this mechanism without a setup period has to be abandoned. Thus, the mechanism with a setup period was chosen as the solution for the dynamic routing mechanism for the IWSN. However, this mechanism is based on an assumption that the topology generated in the setup period is relatively stable which means that the nodes will not frequently change their parent. Next, some experiments will be carried out in order to evaluate the stability of the network.

### 4.3. Stability of Network

**Figure 6:**
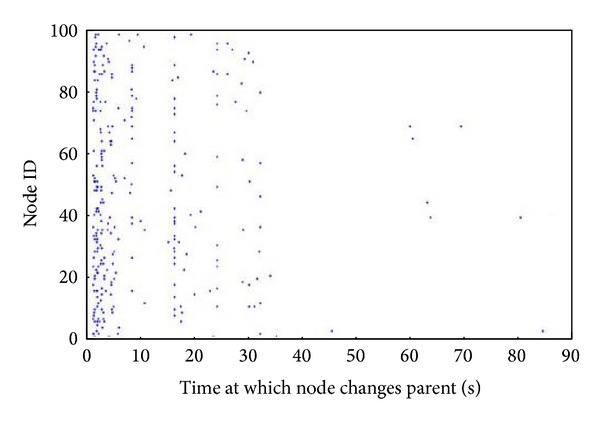
Distribution of the parent changes.

Reasonable values have been obtained for the parameter *τL* and the* ETX_Threshold* from the above simulations. In this experiment, the stability of the network is to be tested when the *τL* is 16 ms and the* ETX_Threshold* is 40. The simulation is run for 90 seconds and the parent changes for each node recorded after the topology has been generated. The result is shown in Figure 6.

As can be seen in Figure 6, the nodes initially change their parents quite frequently but, with the passage of time, the network tends to become stable. Frequent changes occur initially as the nodes discover new links and neighbors and fewer changes once the network has selected high quality routes. However, even initially after the topology has been generated, the number of parent changes is no more than 20 times in 1000 ms. This is quite a small number for a 100-node network.

It is now possible to state that it is possible for a dynamic routing mechanism with a setup period to be applied in an IWSN.

## 5. Efficiency of Dynamic Routing Mechanism

According to the evaluation results obtained in the previous section, it can be determined that the routing mechanism with a setup period can be applied as the dynamic routing mechanism for the TDMA-based IWSN. And the optimal values for the parameters *τL* and* ETX_Threshold* are 16 ms and 40, respectively. The reason is that the time for generating the topology with a reasonable link quality is much too long for a super frame. Another reason is that the topology is relatively stable after it has been generated and thus it would be wasteful to assign a long time for the CSAM period for topology maintenance.

**Figure 7:**
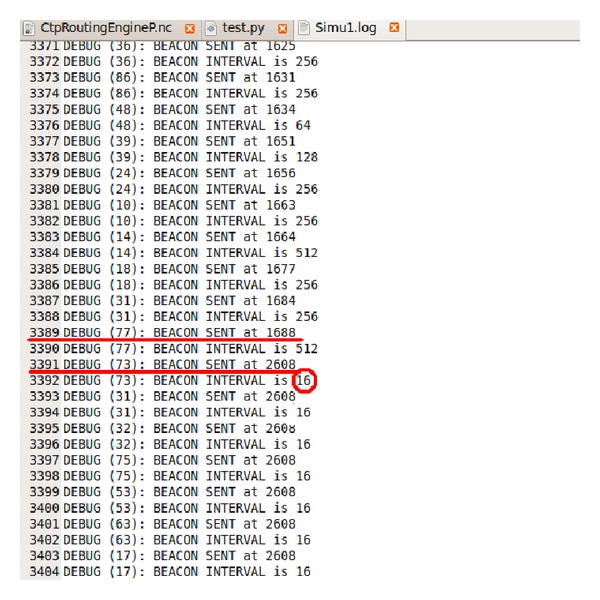
Simulation result of dynamic routing mechanism 1.

**Figure 8:**
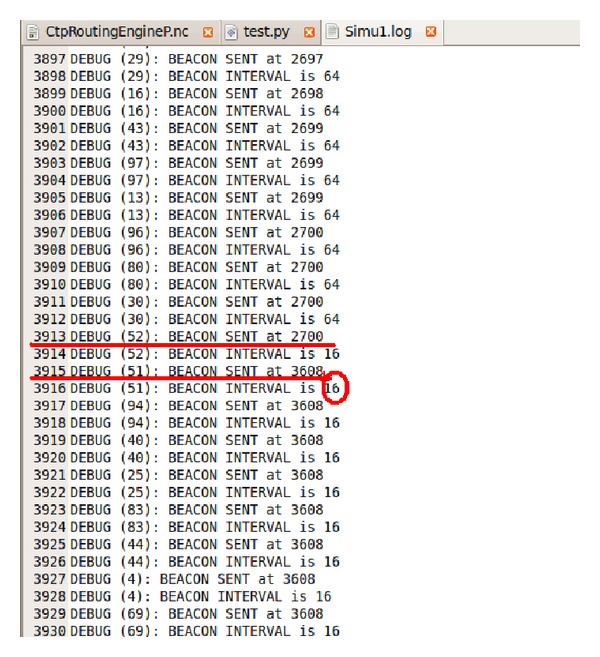
Simulation result of dynamic routing mechanism 2.

The dynamic routing mechanism with a setup period is implemented on TOSSIM and the simulation has been carried out in a network with 100 nodes under the above optimal parameter values. The simulation results are shown in Figures 7 and 8.

As can be seen from Figure 7, the nodes transmit beacon packets in the first 1688 ms which implies that it is in a CSMA stage. After 1688 ms, the nodes stop transmiting beacon packets which implies that it is in a TDMA data transmission stage until 2608 ms. After 2608 ms, the nodes start to transmit beacon packets again which implies that the second super frame arrives. As can be seen from Figure 8, in the second super frame period, the nodes start to transmit beacon packets at 2608 ms and in the CSMA stage. They stop transmiting beacon packets at 2700 ms and enter the TDMA data transmission stage. After 3608 ms the nodes start to transmit beacon packets again which implies that the third super frame arrives. This process loops on and on.

According to the above simulation results, the total time of a super frame *t*_Frame_, the time of the CSMA stage in a super frame *t*_CSMA_, the time of the TDMA stage in a super frame *t*_TDMA_, and the time of the setup period *t*_Setup_ can be calculated as follows:

(5) $$\begin{array}{c} t_{\text{Frame}}=3608-2608=1000\;\text{ms},\\ t_{\text{CSMA}}=2700-2608=92\;\text{ms},\\ t_{\text{TDMA}}=1000-92=908\;\text{ms},\\ t_{\text{Setup}}=1688-92=1596\;\text{ms}. \end{array}$$

Supposing the total number of super frames is *n*, the data transmission efficiency of the dynamic routing mechanism with a setup period *E* can be calculated according to (5) as follows:

(6) $$\begin{array}{c} E=\frac{n*t_{\text{TDMA}}}{t_{\text{Setup}}+n*t_{\text{Frame}}}=\frac{908n}{1596+1000n}. \end{array}$$

**Figure 9:**
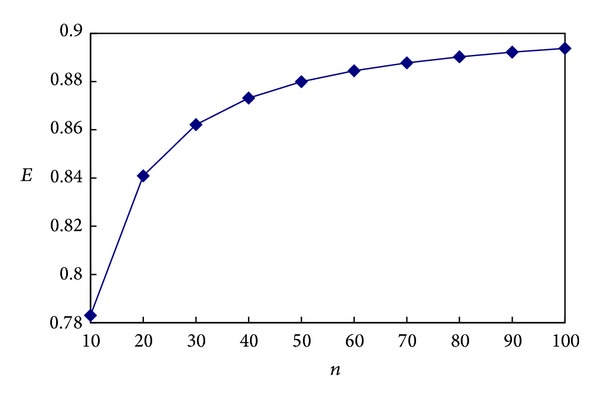
Efficiency of the dynamic routing mechanism.

The number of super frames *n* was set to be 10, 20,…, 100. Therefore the data transmission efficiency can be obtained as in Figure 9.

As can be seen from Figure 9, the data transmission efficiency is always greater than 78% when *n* ≥ 10. With the increase of the number of super frames, the data transmission efficiency increases correspondingly which tends to 90%.

## 6. Conclusions

In this paper, a dynamic routing mechanism was proposed for a TDMA-based industrial wireless sensor network based on the collection tree protocol. The dynamic routing mechanism was evaluated with regard to three aspects: time for generating the topology, link quality, and stability of network through several simulation experiments. The simulation and evaluation results show that this mechanism can act as a solution in relation to the dynamic routing mechanism for the TDMA-based wireless sensor network.

According to the results of the simulation experiments, the time of the setup period of the dynamic routing mechanism has been determined but how much time should be assigned to the CSMA period in each super frame has not. Thus some experiments should be designed and conducted to determine the time of CSMA period in each super frame in the future.

## Figures and Tables

**Table 1:** Time of generating topology with different *ETX_Threshold* values.

*ETX_Threshold* value	Time of generating topology (ms)
50	1176
40	1529
30	16380
20	280000
